# Supplementary material for: E-commerce recommender system design based on web information extraction and sentiment analysis
Source: PLoS One. 2025 Sep 23;20(9):e0327213. doi: 10.1371/journal.pone.0327213 (PMC12456782; doi:10.1371/journal.pone.0327213)
Supplement: Attachment — Submitted filename: E-commerce Recommender System Design.pdf [file pone.0327213.s001.pdf]

Your manuscript provides a broad exploration of "**E-commerce Recommender System Design Based on Web Information Extraction and Sentiment Analysis"**. While the paper addresses significant aspects of web information extraction and sentiment analysis for E-commerce recommender system, some areas require revision to enhance clarity, coherence, and depth of analysis.

1. Your title and explanation mechanism are well-crafted and clear. However, some abbreviations are used throughout the document without being fully defined.
2. The originality of the work is quite restricted, and the literature review section is insufficient, lacking state-of-the-art and recent studies. A significant expansion and elaboration of the review of related literature is necessary, incorporating the highest quality relevant research. It is crucial to clearly highlight the gaps in the current knowledge base and explain how this study has effectively addressed or filled these gaps.
3. Your contribution is not clearly articulated; could you clarify what it is?
4. To make recommendations for e-commerce users, what types of data did you use? I believe you collected user comments. If so, do these comments contribute to recommendations for e-commerce features?
5. The Results section should be updated by comparing your result to the other work which is stated under related work.
6. The conclusion needs to be updated with the proposed methodology result and the future scope of the work.
