# Supplementary material for: E-commerce recommender system design based on web information extraction and sentiment analysis
Source: PLoS One. 2025 Sep 23;20(9):e0327213. doi: 10.1371/journal.pone.0327213 (PMC12456782; doi:10.1371/journal.pone.0327213)
Supplement: Attachment — Submitted filename: Author response.docx [file pone.0327213.s004.docx]

Review Comments to the Author

**Reviewer #1:**

1.The paper mentions multi-dimensional emotion analysis and its potential for exploring emotion trend prediction and reason analysis in the introduction, but it does not provide a detailed explanation or exploration of these aspects in the methodology, experiments, or discussion.
Reply: Thank you for your question, the aspects of multidimensional emotion analysis have been explained and explored in detail in methodology, experiments and discussions. Related changes are as follows:

In order to further explore the emotional trend and the reasons behind the emotion in the e-commerce recommendation system, the study introduces the affective trend prediction. The time series analysis method is used to model the sentiment value of user comments. First, the user comments were sorted chronologically and the sentiment score for each point in time was calculated. Then, sliding window technology is used to smooth the emotion score to reduce the impact of short-term fluctuations. Then, autoregressive moving average (ARMA) model or long short-term memory network (LSTM) were used to model the time series of emotion scores. By training the model, it is possible to predict emotional trends at future points in time, thus providing early warning for businesses to adjust product strategies or marketing programs in a timely manner. The purpose of the analysis of the reasons behind emotion is to explore the potential factors of the user's emotional tendency. Therefore, the method of causality analysis is adopted. First, text mining techniques are used to extract emotion-related keywords and phrases from user reviews that may be related to specific attributes of the product (such as price, quality, features, etc.). Then, the association rule mining algorithm is used to analyze the association relationship between these keywords and phrases to identify the factors that may cause the emotion change.

In order to verify the performance advantages of the e-commerce recommendation system proposed in this paper in multidimensional emotion analysis, especially the ability to predict emotional trends and analyze the reasons behind emotions, two sets of experiments were designed and compared with the current most advanced e-commerce recommendation system (the system used by Amazon (Patil P et al. (2024)) and Netflix (Singh R et al. (2024))), as shown in Table 2.

**Table 2 Performance comparison of multidimensional sentiment analysis of e-commerce recommendation systems**

| Experimental index | Our system | Amazon recommendation system | Netflix recommendation system |
| --- | --- | --- | --- |
| Mean square error (MSE) | 0.035 | 0.052 | 0.048 |
| Mean absolute error (MAE) | 0.021 | 0.033 | 0.030 |
| Prediction accuracy | 92.5% | 88.0% | 89.5% |
| Model training time (seconds) | 120 | 180 | 150 |
| Association rule confidence | 0.85 | 0.78 | 0.80 |
| Association rule support | 0.60 | 0.52 | 0.55 |
| Affective cause analysis accuracy | 90.0% | 85.0% | 87.0% |
| Analysis time (seconds) | 90 | 120 | 110 |

In Table 2, the MSE of the proposed system is 0.035, which is much lower than Amazon's 0.052 and Netflix's 0.048, indicating that this system has obvious advantages in the accuracy of emotional trend prediction. At the same time, the MAE of this system is 0.021, which is lower than Amazon's 0.033 and Netflix's 0.030, which further proves its superiority in forecasting accuracy. In addition, the system's prediction accuracy reached 92.5%, higher than Amazon's 88.0% and Netflix's 89.5%, which indicates that the system can more accurately predict the changing trend of user emotion. The association rule confidence of the system is 0.85, which is higher than Amazon's 0.78 and Netflix's 0.80, indicating that the system can more accurately identify the key factors behind the emotion. At the same time, the association rule support of this system is 0.60, which is also higher than Amazon's 0.52 and Netflix's 0.55, indicating that this system has advantages in the universality and reliability of emotional reason analysis. In addition, the accuracy rate of emotional reason analysis of this system reaches 90.0%, which is higher than that of Amazon (85.0%) and Netflix (87.0%), which further proves its superiority in emotional reason analysis. Although the analysis time of this system is slightly shorter than that of Amazon and Netflix, its significant improvement in analysis accuracy and reliability indicates that this system has higher practicality and accuracy in analyzing the reasons behind emotions.

The system in this paper performs well in the two key tasks of emotion trend prediction and emotion reason analysis, and its prediction accuracy and analysis accuracy are better than the advanced recommendation system used by Amazon and Netflix. This indicates that the e-commerce recommendation system proposed in this paper has significant advantages in multidimensional emotion analysis, which can provide users with more accurate and personalized recommendation services, and also provide merchants with more in-depth user emotion insight.

2. The paper tackles the idea of ecommerce recommendation system but doesn't provide any benchmark against state-of-the-art e-commerce recommender systems like those used by Amazon or Netflix.

Reply: Thank you for your suggestion. Benchmark experiments compared to state-of-the-art e-commerce recommendation systems used by Amazon or Netflix have been supplemented. Please see the details below:

In order to verify the performance advantages of the e-commerce recommendation system proposed in this paper in multidimensional emotion analysis, especially the ability to predict emotional trends and analyze the reasons behind emotions, two sets of experiments were designed and compared with the current most advanced e-commerce recommendation system (the system used by Amazon (Patil P et al. (2024)) and Netflix (Singh R et al. (2024))), as shown in Table 2.

**Table 2 Performance comparison of multidimensional sentiment analysis of e-commerce recommendation systems**

| Experimental index | Our system | Amazon recommendation system | Netflix recommendation system |
| --- | --- | --- | --- |
| Mean square error (MSE) | 0.035 | 0.052 | 0.048 |
| Mean absolute error (MAE) | 0.021 | 0.033 | 0.030 |
| Prediction accuracy | 92.5% | 88.0% | 89.5% |
| Model training time (seconds) | 120 | 180 | 150 |
| Association rule confidence | 0.85 | 0.78 | 0.80 |
| Association rule support | 0.60 | 0.52 | 0.55 |
| Affective cause analysis accuracy | 90.0% | 85.0% | 87.0% |
| Analysis time (seconds) | 90 | 120 | 110 |

In Table 2, the MSE of the proposed system is 0.035, which is much lower than Amazon's 0.052 and Netflix's 0.048, indicating that this system has obvious advantages in the accuracy of emotional trend prediction. At the same time, the MAE of this system is 0.021, which is lower than Amazon's 0.033 and Netflix's 0.030, which further proves its superiority in forecasting accuracy. In addition, the system's prediction accuracy reached 92.5%, higher than Amazon's 88.0% and Netflix's 89.5%, which indicates that the system can more accurately predict the changing trend of user emotion. The association rule confidence of the system is 0.85, which is higher than Amazon's 0.78 and Netflix's 0.80, indicating that the system can more accurately identify the key factors behind the emotion. At the same time, the association rule support of this system is 0.60, which is also higher than Amazon's 0.52 and Netflix's 0.55, indicating that this system has advantages in the universality and reliability of emotional reason analysis. In addition, the accuracy rate of emotional reason analysis of this system reaches 90.0%, which is higher than that of Amazon (85.0%) and Netflix (87.0%), which further proves its superiority in emotional reason analysis. Although the analysis time of this system is slightly shorter than that of Amazon and Netflix, its significant improvement in analysis accuracy and reliability indicates that this system has higher practicality and accuracy in analyzing the reasons behind emotions.

**Reviewer #2:**

Your manuscript provides a broad exploration of “E-commerce Recommender System Design Based on Web Information Extraction and Sentiment Analysis". While the paper addresses significant aspects of web information extraction and sentiment analysis for E-commerce recommender system, some areas require revision to enhance clarity, coherence, and depth of analysis.

1. Your title and explanation mechanism are well-crafted and clear. However, some abbreviations are used throughout the document without being fully defined.

Reply: Thank you for your question, all abbreviations in the definition manuscript have been thoroughly checked. Please see the details below:

Electromyography (EMG)

Electromyography-Hand (EMGH)

2. The originality of the work is quite restricted, and the literature review section is insufficient, lacking state-of-the-art and recent studies. A significant expansion and elaboration of the review of related literature is necessary, incorporating the highest quality relevant research. It is crucial to clearly highlight the gaps in the current knowledge base and explain how this study has effectively addressed or filled these gaps.

Reply: Thank you for your suggestion. The literature review section has been expanded to include relevant research of the highest quality, highlighting gaps in the current knowledge base and explaining how this study effectively addresses or fills those gaps. As follows:

In view of the problems of sparse data and changing user preferences faced by traditional collaborative filtering algorithms in online commerce, Patil P et al. (2024) proposed a recommendation system method that combines collaborative filtering and content-based filtering. The research results show that the system provides accurate recommendations for new and old users by analyzing user purchasing behavior and feedback. In view of the limitations of traditional collaborative filtering methods in multi-criteria recommendation systems, such as the shortcomings of single-standard collaborative filtering (CF) in dealing with cold start problems and related-based similarity problems, Singh R et al. (2024) compared and evaluated traditional collaborative filtering, matrix decomposition and deep matrix decomposition techniques. The results show that the deep matrix decomposition technique performs better than other traditional methods on multi-criteria data sets and can provide more accurate recommendations.

Patil P, Kadam S U, Aruna E R, More A, Balajee R M, Rao B N K. Recommendation System for E-Commerce Using Collaborative Filtering. Journal Europeen des Systemes Automatises, 2024, 57(4):1145-1153.

Singh R, Dwivedi P, Kant V . Comparative analysis of collaborative filtering techniques for the multi?criteria recommender systems. Multimedia tools and applications, 2024, 83(24):64551-64571.

To sum up, in the field of information extraction, although various algorithms have been proposed to improve the efficiency and accuracy of data capture, they still face the challenges of data sparsity and noise interference when processing large-scale and dynamically changing web data. In terms of emotion analysis, although existing studies can classify consumers' emotional tendency, they are still insufficient in multidimensional emotion analysis, emotional trend prediction and in-depth exploration of the reasons behind emotions. In addition, in the field of recommendation system, the traditional collaborative filtering method has limitations in dealing with the cold start problem of new users and multi-standard recommendation, and the existing improved methods have not fully solved these problems. The research aims to fill these gaps by integrating information extraction, sentiment analysis and recommendation system techniques. Through the improved S-PageRank algorithm and the dynamic topic library generation strategy, the accuracy and efficiency of cross-platform information extraction are improved, and the problems of data sparsity and noise interference are solved. Secondly, combining topic model and deep learning technology, a bidirectional short-duration memory network and comprehensive attention mechanism model integrating theme word features are proposed to achieve more accurate multidimensional emotion analysis and emotion trend prediction. Finally, by combining collaborative filtering and content-based filtering methods, an intelligent recommendation system is designed to provide personalized recommendations for new and old users.

3. Your contribution is not clearly articulated; could you clarify what it is?

Reply: Thank you for your question. Contributions to research have been supplemented. See below:

The contribution of the research is to propose a web page information extraction method combining the S-PageRank algorithm of keyword weight allocation and the dynamic topic library generation strategy, which effectively improves the accuracy and accuracy of the topic crawler, and solves the problems of "local optimal solution" and "topic deviation" which are easy to appear in the traditional algorithm in the massive web pages. The introduction of template based web page information automatic extraction strategy, through the semantic annotation of DOM tree and template matching, the rapid and accurate extraction of commodity information is realized, and the information extraction time is greatly shortened.

4. To make recommendations for e-commerce users, what types of data did you use? I believe you collected user comments. If so, do these comments contribute to recommendations for e-commerce features?

Reply: Thank you for your suggestion. The data type used has been added. It has been explained that user reviews contribute to the recommendation of e-commerce features. Related changes are as follows:

In making recommendations for e-commerce users, the research mainly uses the following types of data. The first is user review data, through the analysis of users' comments on goods, in-depth understanding of users' satisfaction, preferences and potential demand for goods. The second is the user behavior data, which includes: purchase history, the user's purchase record reflects its actual purchase behavior and preferences. Browsing behavior: The user's browsing history (such as clicks, time spent, etc.) reveals the user's level of interest in certain products. Search history: Users' search keywords can reflect their current needs and interests. The third is the commodity attribute data, including the detailed description, function, characteristics and other information of the commodity, which can help the recommendation system to better understand the characteristics of the commodity, so as to achieve content-based recommendation. It also includes product classification information (such as electronics, clothing, household goods, etc.), which is used to build a similarity relationship between products and help the recommendation system find products that are similar to the user's historical behavior. Price information: Product price ranges and promotions are used to meet users' budget needs and provide more cost-effective recommendations. The fourth is user profile data, including demographic information, interest labels, and social network data. User reviews play an important role in e-commerce recommendation function, which can not only improve the accuracy and personalization of recommendations, but also enhance user engagement and user experience of the platform.

5. The Results section should be updated by comparing your result to the other work which is stated under related work.

Reply: Thank you for your question. Results have been compared with other work described under related work. See below:

In order to verify the performance advantages of the e-commerce recommendation system proposed in this paper in multidimensional emotion analysis, especially the ability to predict emotional trends and analyze the reasons behind emotions, two sets of experiments were designed and compared with the current most advanced e-commerce recommendation system (the system used by Amazon (Patil P et al. (2024)) and Netflix (Singh R et al. (2024))), as shown in Table 2.

**Table 2 Performance comparison of multidimensional sentiment analysis of e-commerce recommendation systems**

| Experimental index | Our system | Amazon recommendation system | Netflix recommendation system |
| --- | --- | --- | --- |
| Mean square error (MSE) | 0.035 | 0.052 | 0.048 |
| Mean absolute error (MAE) | 0.021 | 0.033 | 0.030 |
| Prediction accuracy | 92.5% | 88.0% | 89.5% |
| Model training time (seconds) | 120 | 180 | 150 |
| Association rule confidence | 0.85 | 0.78 | 0.80 |
| Association rule support | 0.60 | 0.52 | 0.55 |
| Affective cause analysis accuracy | 90.0% | 85.0% | 87.0% |
| Analysis time (seconds) | 90 | 120 | 110 |

In Table 2, the MSE of the proposed system is 0.035, which is much lower than Amazon's 0.052 and Netflix's 0.048, indicating that this system has obvious advantages in the accuracy of emotional trend prediction. At the same time, the MAE of this system is 0.021, which is lower than Amazon's 0.033 and Netflix's 0.030, which further proves its superiority in forecasting accuracy. In addition, the system's prediction accuracy reached 92.5%, higher than Amazon's 88.0% and Netflix's 89.5%, which indicates that the system can more accurately predict the changing trend of user emotion. The association rule confidence of the system is 0.85, which is higher than Amazon's 0.78 and Netflix's 0.80, indicating that the system can more accurately identify the key factors behind the emotion. At the same time, the association rule support of this system is 0.60, which is also higher than Amazon's 0.52 and Netflix's 0.55, indicating that this system has advantages in the universality and reliability of emotional reason analysis. In addition, the accuracy rate of emotional reason analysis of this system reaches 90.0%, which is higher than that of Amazon (85.0%) and Netflix (87.0%), which further proves its superiority in emotional reason analysis. Although the analysis time of this system is slightly shorter than that of Amazon and Netflix, its significant improvement in analysis accuracy and reliability indicates that this system has higher practicality and accuracy in analyzing the reasons behind emotions.

6. The conclusion needs to be updated with the proposed methodology result and the future scope of the work.

Reply: Thank you for your suggestion. The results in the conclusions and the scope of future work have been updated. As follows:

The system in this paper performs well in the two key tasks of emotion trend prediction and emotion reason analysis, and its prediction accuracy and analysis accuracy are better than the advanced recommendation system used by Amazon and Netflix. This indicates that the e-commerce recommendation system proposed in this paper has significant advantages in multidimensional emotion analysis, which can provide users with more accurate and personalized recommendation services, and also provide merchants with more in-depth user emotion insight.

Future work will focus on further optimizing the recommendation algorithm to improve the accuracy and personalization of recommendations by introducing more fine-grained sentiment analysis and multi-dimensional user behavior modeling. In addition, the research will explore how to better integrate real-time user feedback and social network data to enhance the dynamic adaptability and user interaction of recommendation systems.
