# Supplementary material for: E-commerce recommender system design based on web information extraction and sentiment analysis
Source: PLoS One. 2025 Sep 23;20(9):e0327213. doi: 10.1371/journal.pone.0327213 (PMC12456782; doi:10.1371/journal.pone.0327213)
Supplement: Attachment — Submitted filename: Revisions.docx [file pone.0327213.s003.docx]

I found the subject of study quite fascinating. It is quite striking. Congratulations to the researchers. However, the study needs to be improved in terms of the following issues.

1. There is unnecessary information in the abstract, there is nothing necessary. We expect a scientific manuscript presenting the results of the study, not a report summarizing a study.
2. The success achieved in the study and the reasons why this success was achieved should be explained in detail in a paragraph. Its superiority over similar studies must be demonstrated.
3. The organization and structure of the article should be revised to make the subject more understandable.
4. Authors are advised to read the latest citations as per the scope of the paper. I am suggesting some related citations to enhance the quality of research paper. The references are as follows:

1.	Zuo, C., Zhang, X., Yan, L., & Zhang, Z. (2024). GUGEN: Global User Graph Enhanced Network for Next POI Recommendation. IEEE Transactions on Mobile Computing, 23(12), 14975-14986. doi: 10.1109/TMC.2024.3455107

2.	Yang, K. (2024). How to prevent deception: A study of digital deception in "visual poverty" livestream. New Media & Society. doi: 10.1177/14614448241285443

3.	Wu, L., Long, Y., Gao, C., Wang, Z., & Zhang, Y. (2023). MFIR: Multimodal fusion and inconsistency reasoning for explainable fake news detection. Information Fusion, 100, 101944. doi: https://doi.org/10.1016/j.inffus.2023.101944

4.	Song, L., Chen, S., Meng, Z., Sun, M., & Shang, X. (2024). FMSA-SC: A Fine-Grained Multimodal Sentiment Analysis Dataset Based on Stock Comment Videos. IEEE Transactions on Multimedia, 26, 7294-7306. doi: 10.1109/TMM.2024.3363641

5.	Wang, E., Song, Z., Wu, M., Liu, W., Yang, B., Yang, Y.,... Wu, J. (2025). A New Data Completion Perspective on Sparse CrowdSensing: Spatiotemporal Evolutionary Inference Approach. IEEE Transactions on Mobile Computing, 24(3), 1357-1371. doi: 10.1109/TMC.2024.3480983

6.	Xu, Y., Zhuang, F., Wang, E., Li, C., & Wu, J. (2025). Learning Without Missing-At-Random Prior Propensity-A Generative Approach for Recommender Systems. IEEE Transactions on Knowledge and Data Engineering, 37(2), 754-765. doi: 10.1109/TKDE.2024.3490593

7.	Lin, X., Liu, R., Cao, Y., Zou, L., Li, Q., Wu, Y.,... Xu, G. (2025). Contrastive Modality-Disentangled Learning for Multimodal Recommendation. ACM Trans. Inf. Syst., 43(3), 70. doi: 10.1145/3715876

8.	Shi, J., Liu, C., & Liu, J. (2024). Hypergraph-Based Model for Modeling Multi-Agent Q-Learning Dynamics in Public Goods Games. IEEE Transactions on Network Science and Engineering, 11(6), 6169-6179. doi: 10.1109/TNSE.2024.3473941

9.	Zhu, C. (2023). Research on Emotion Recognition-Based Smart Assistant System: Emotional Intelligence and Personalized Services. Journal of System and Management Sciences, 13(5), 227-242. doi: 10.33168/JSMS.2023.0515

10.	Deng, Q., Chen, X., Lu, P., Du, Y., & Li, X. (2025). Intervening in Negative Emotion Contagion on Social Networks Using Reinforcement Learning. IEEE Transactions on Computational Social Systems, 1-12. doi: 10.1109/TCSS.2025.3555607

11.	Li, T., Li, Y., Zhang, M., Tarkoma, S., & Hui, P. (2023). You Are How You Use Apps: User Profiling Based on Spatiotemporal App Usage Behavior. ACM Trans. Intell. Syst. Technol., 14(4). doi: 10.1145/3597212

12.	Li, T., Li, Y., Xia, T., & Hui, P. (2021). Finding Spatiotemporal Patterns of Mobile Application Usage. IEEE Transactions on Network Science and Engineering. doi: 10.1109/TNSE.2021.3131194

13.	Ding, J., Chen, X., Lu, P., Yang, Z., Li, X.,... Du, Y. (2023). DialogueINAB: an interaction neural network based on attitudes and behaviors of interlocutors for dialogue emotion recognition. The Journal of Supercomputing, 79(18), 20481-20514. doi: 10.1007/s11227-023-05439-1

Overall, there are still some minor parts that the authors did not explain clearly. Some additional evaluations are expected to be in the manuscript as well. As a result, I am going to suggest Minor revision of the paper in its present form.
