# Supplementary material for: E-commerce recommender system design based on web information extraction and sentiment analysis
Source: PLoS One. 2025 Sep 23;20(9):e0327213. doi: 10.1371/journal.pone.0327213 (PMC12456782; doi:10.1371/journal.pone.0327213)
Supplement: Attachment — Submitted filename: Response to reviewers comments.doc [file pone.0327213.s005.doc]

Journa l Requirements:

Please review your reference list to ensure that it is complete and correct. If you have cited papers t hat have been retracted, please include the rationale for doing so in the manuscript text, or remove these references and replace them with relevant current references. Any changes to the reference list should be mentioned in the rebuttal letter that accompanies your revised manuscript. If you need to cite a retracted article, indicate the article’s retracted status in the References list and also include a citation and full reference for the retraction notice.

Reviewer #2: The author addressd the previous comments. The pa per proposed smart recommendation system and better to compare the performance of your system with other systems with the same parameters and algorithms.

Reply: Thank you for your recognition.

Reviewer #3: I found the subject of study quite fascinating. It is quite striking. Congratulations to the researchers. However, the study needs to be improved in terms of the following issues.
1. There is unnecessary information in the abstract, there is nothing necessary. We expect a scientific manuscript presenting the results of the study, not a report summarizing a study.
Reply: Thank you for your question. The abstract has been rewritten, focusing more on presenting the research results rather than summarizing the research process. As follows:

The research proposes an e-commerce recommendation system based on web page information extraction and sentiment analysis. Through the improved S-PageRank algorithm and the dynamic topic library generation strategy, the precision rate of cross-platform commodity information extraction has been significantly improved to 90%, which is superior to the traditional S-PageRank algorithm. The template-based web page information extraction method performs well, with a precision rate 10% higher than that of the method based on the document object model. In terms of sentiment analysis, the comprehensive attention mechanism model combining the topic model and the bidirectional long short-term memory network has achieved the precise calculation of the sentiment scores of each topic in the customer evaluation. When the number of topics of the LDA model is 7, the prediction accuracy reaches its peak, and the model outperforms previous methods in terms of accuracy, recall rate and F-score. The experimental results show that this recommendation system performs excellently in the prediction of sentiment trends and the analysis of the reasons behind emotions. Its prediction accuracy and analysis accuracy are both superior to existing recommendation systems such as Amazon and Netflix. This system can provide users with more accurate and personalized product recommendation services, and at the same time offer merchants deeper insights into users' emotions.

2. The success achieved in the study and the reasons why this success was achieved should be explained in detail in a paragraph. Its superiority over similar studies must be demonstrated.
Reply: Thank you for your suggestion. A discussion chapter has been added, which elaborates in detail on the success achieved in the research and the reasons for its success, demonstrating its superiority over similar studies. The revisions were shown below:

**5. Discussion**

The research has achieved remarkable results in cross-platform e-commerce web page information extraction and sentiment analysis, especially excelling in precision rate, the accuracy of sentiment analysis and the overall performance of the recommendation system. The S-PageRank algorithm based on keyword weight allocation and the dynamic topic library generation strategy proposed in the research have effectively improved the precision rate of topic crawlers. The precision rate of this method has reached 90%, which is significantly better than the traditional S-PageRank algorithm. Compared with the research of Li et al., this study has significantly improved in both the accuracy and efficiency of information extraction. Although Luscombe et al. emphasized the importance of web scraping, when dealing with large-scale dynamic web page data, their methods still face the challenges of data sparsity and noise interference (Li et al., 2021). The research effectively solved these problems by optimizing the algorithm and introducing the template strategy. When the number of topics of the LDA model is 7, the prediction accuracy of the model reaches its peak, and it is superior to the previous methods in terms of accuracy, recall rate and F-score. The research introduces a comprehensive attention mechanism, taking into account not only the word positions in the comment texts but also the contribution degrees of different parts to the sentiment tendencies, thereby calculating the sentiment values of user comments under different topics more accurately. The MSE of the e-commerce recommendation system proposed in the study is 0.035, the MAE is 0.021, and the prediction accuracy rate reaches 92.5%, all of which are superior to the existing recommendation systems such as Amazon and Netflix. Furthermore, the confidence level and support level of the association rules of the system are also higher than those of Amazon and Netflix, indicating that it has significant advantages in the universality and reliability of sentiment cause analysis. Compared with the recommendation system method of integrated collaborative filtering and content-based filtering proposed by Ding et al., the recommendation system in this study not only performs well in dealing with the cold start problem of new users, but also provides more accurate personalized services in multi-criterion recommendation. Although Patil et al. 's method provides precise recommendations for users by analyzing their purchasing behaviors and feedback, it still faces challenges when dealing with large-scale dynamic data (Ding et al., 2023). The research fills these gaps by comprehensively applying information extraction, sentiment analysis and recommendation system technologies.

3. The organization and structure of the article should be revised to make the subject more understandable.
Reply: Thank you for your question. The organizational structure of the article has been adjusted and discussion chapters have been added.

4. Authors are advised to read the latest citations as per the scope of the paper. I am suggesting some related citations to enhance the quality of research paper. The references are as follows:
1. Zuo, C., Zhang, X., Yan, L., & Zhang, Z. (2024). GUGEN: Global User Graph Enhanced Network for Next POI Recommendation. IEEE Transactions on Mobile Computing, 23(12), 14975-14986. doi: 10.1109/TMC.2024.3455107
2. Yang, K. (2024). How to prevent deception: A study of digital deception in "visual poverty" livestream. New Media & Society. doi: 10.1177/14614448241285443
3. Wu, L., Long, Y., Gao, C., Wang, Z., & Zhang, Y. (2023). MFIR: Multimodal fusion and inconsistency reasoning for explainable fake news detection. Information Fusion, 100, 101944. doi: https://doi.org/10.1016/j.inffus.2023.101944
4. Song, L., Chen, S., Meng, Z., Sun, M., & Shang, X. (2024). FMSA-SC: A Fine-Grained Multimodal Sentiment Analysis Dataset Based on Stock Comment Videos. IEEE Transactions on Multimedia, 26, 7294-7306. doi: 10.1109/TMM.2024.3363641
5. Wang, E., Song, Z., Wu, M., Liu, W., Yang, B., Yang, Y.,... Wu, J. (2025). A New Data Completion Perspective on Sparse CrowdSensing: Spatiotemporal Evolutionary Inference Approach. IEEE Transactions on Mobile Computing, 24(3), 1357-1371. doi: 10.1109/TMC.2024.3480983
6. Xu, Y., Zhuang, F., Wang, E., Li, C., & Wu, J. (2025). Learning Without Missing-At-Random Prior Propensity-A Generative Approach for Recommender Systems. IEEE Transactions on Knowledge and Data Engineering, 37(2), 754-765. doi: 10.1109/TKDE.2024.3490593
7. Lin, X., Liu, R., Cao, Y., Zou, L., Li, Q., Wu, Y.,... Xu, G. (2025). Contrastive Modality-Disentangled Learning for Multimodal Recommendation. ACM Trans. Inf. Syst., 43(3), 70. doi: 10.1145/3715876
8. Shi, J., Liu, C., & Liu, J. (2024). Hypergraph-Based Model for Modeling Multi-Agent Q-Learning Dynamics in Public Goods Games. IEEE Transactions on Network Science and Engineering, 11(6), 6169-6179. doi: 10.1109/TNSE.2024.3473941
9. Zhu, C. (2023). Research on Emotion Recognition-Based Smart Assistant System: Emotional Intelligence and Personalized Services. Journal of System and Management Sciences, 13(5), 227-242. doi: 10.33168/JSMS.2023.0515
10. Deng, Q., Chen, X., Lu, P., Du, Y., & Li, X. (2025). Intervening in Negative Emotion Contagion on Social Networks Using Reinforcement Learning. IEEE Transactions on Computational Social Systems, 1-12. doi: 10.1109/TCSS.2025.3555607
11. Li, T., Li, Y., Zhang, M., Tarkoma, S., & Hui, P. (2023). You Are How You Use Apps: User Profiling Based on Spatiotemporal App Usage Behavior. ACM Trans. Intell. Syst. Technol., 14(4). doi: 10.1145/3597212
12. Li, T., Li, Y., Xia, T., & Hui, P. (2021). Finding Spatiotemporal Patterns of Mobile Application Usage. IEEE Transactions on Network Science and Engineering. doi: 10.1109/TNSE.2021.3131194
13. Ding, J., Chen, X., Lu, P., Yang, Z., Li, X.,... Du, Y. (2023). DialogueINAB: an interaction neural network based on attitudes and behaviors of interlocutors for dialogue emotion recognition. The Journal of Supercomputing, 79(18), 20481-20514. doi: 10.1007/s11227-023-05439-1

Reply: Thank you for your suggestion. The references you recommended have been cited in the paper. Related changes are as follows:

Zuo C, Zhang X, Yan L, Zhang Z. GUGEN: Global User Graph Enhanced Network for Next POI Recommendation . IEEE Transactions on Mobile Computing, 2024, 23(12): 14975-14986.

Yang K. How to prevent deception: A study of digital deception in "visual poverty" livestream . New Media & Society, 2024.

Wu L, Long Y, Gao C, Wang Z, Zhang Y. MFIR: Multimodal fusion and inconsistency reasoning for explainable fake news detection . Information Fusion, 2023, 100: 101944.

Song L, Chen S, Meng Z, Sun M, Shang X. FMSA-SC: A Fine-Grained Multimodal Sentiment Analysis Dataset Based on Stock Comment Videos . IEEE Transactions on Multimedia, 2024, 26: 7294-7306.

Wang E, Song Z, Wu M, Liu W, Yang B, Yang Y, et al. A New Data Completion Perspective on Sparse CrowdSensing: Spatiotemporal Evolutionary Inference Approach . IEEE Transactions on Mobile Computing, 2025, 24(3): 1357-1371.

Xu Y, Zhuang F, Wang E, Li C, Wu J. Learning Without Missing-At-Random Prior Propensity-A Generative Approach for Recommender Systems . IEEE Transactions on Knowledge and Data Engineering, 2025, 37(2): 754-765.

Lin X, Liu R, Cao Y, Zou L, Li Q, Wu Y, et al. Contrastive Modality-Disentangled Learning for Multimodal Recommendation . ACM Trans. Inf. Syst., 2025, 43(3): 70.

Shi J, Liu C, Liu J. Hypergraph-Based Model for Modeling Multi-Agent Q-Learning Dynamics in Public Goods Games . IEEE Transactions on Network Science and Engineering, 2024, 11(6): 6169-6179.

Zhu C. Research on Emotion Recognition-Based Smart Assistant System: Emotional Intelligence and Personalized Services . Journal of System and Management Sciences, 2023, 13(5): 227-242.

Deng Q, Chen X, Lu P, Du Y, Li X. Intervening in Negative Emotion Contagion on Social Networks Using Reinforcement Learning . IEEE Transactions on Computational Social Systems, 2025: 1-12.

Li T, Li Y, Zhang M, Tarkoma S, Hui P. You Are How You Use Apps: User Profiling Based on Spatiotemporal App Usage Behavior . ACM Trans. Intell. Syst. Technol., 2023, 14(4).

Li T, Li Y, Xia T, Hui P. Finding Spatiotemporal Patterns of Mobile Application Usage . IEEE Transactions on Network Science and Engineering, 2021.

Ding J, Chen X, Lu P, Yang Z, Li X, et al. DialogueINAB: an interaction neural network based on attitudes and behaviors of interlocutors for dialogue emotion recognition . The Journal of Supercomputing, 2023, 79(18): 20481-20514.

Overall, there are still some minor parts that the authors did not explain clearly. Some additional evaluations are expected to be in the manuscript as well. As a result, I am going to suggest Minor revision of the paper in its present form.

Reply: Thank you for your question. The manuscript has been carefully revised in accordance with the questions you raised.
